# Editorial: Coping With Environmental Fluctuations: Ecological and Evolutionary Perspectives

**DOI:** 10.3389/fphys.2020.605186

**Published:** 2020-10-23

**Authors:** Sylvain Giroud, Andreas Nord, Kenneth B. Storey, Julia Nowack

**Affiliations:** ^1^Research Institute of Wildlife Ecology, Department of Interdisciplinary Life Sciences, University of Veterinary Medicine Vienna, Vienna, Austria; ^2^Section for Evolutionary Ecology, Department of Biology, Lund University, Lund, Sweden; ^3^Department of Biology, Carleton University, Ottawa, ON, Canada; ^4^School of Biological and Environmental Sciences, Liverpool John Moores University, Liverpool, United Kingdom

**Keywords:** climate change, phenotypic flexibility, endotherm, ectotherm, heterothermy, hypoxia, thermoregulation, seasonality

Many organisms are adapted to endure substantial seasonal fluctuations in one or several environmental parameters. Recently, organisms across the globe also have to deal with accelerated climate change that is characterized by both a slow increase in mean temperature and increased frequency of extreme weather events (IPCC, 2013). To predict how this will affect wildlife, there is a need to understand how ecology and physiology have evolved to match the demands of an animal's current range. This Research Topic is comprised of both primary research and reviews that provide a comprehensive overview of recent advances in mechanistic and eco-evolutionary aspects of how animals cope with changing environments. Studies range over molecular, cellular, and organismal levels of enquiry and from individual animals to populations of mammals, birds, and selected ectotherms. Collectively, the Research Topic covers three main aspects of how animals respond to environmental fluctuations: (i) seasonal expression and evolution of hypometabolism and heterothermy; (ii) metabolic responses to climate change in dry, energy-limited, or hypoxic environments; and (iii) thermal sensitivity of individuals during development and its potential lifelong effects.

To face seasonal declines in resource abundance and/or temperature, many endothermic animals have developed the ability to save energy via heterothermy, whereby metabolic rate is substantially reduced and body temperature falls to within a few degrees of ambient (Ruf and Geiser, 2015). In his review, Geiser outlines the state-of-the-art of ecological, physiological, and life-history correlates of heterothermy. He remarks that hibernation (i.e., hypometabolic states lasting days or weeks), which occurs in mammals from all three subclasses but only in a single bird species, is not as seasonal as commonly thought and is strictly limited to winter in relatively few species. The expression of short bouts of daily torpor (i.e., hypometabolic states lasting a few hours) varies depending on environmental conditions and predation pressure. For this reason, the seasonal expression of torpor is not as clear-cut as commonly assumed and differs between true hibernators and daily heterotherms. Torpor is often associated with cold and seasonal habitats, but it is also common in the tropics and elsewhere at low latitudes. Nowack et al. report that the triggers of heterothermy and resulting patterns of metabolic rate and body temperature in low latitude heterotherms are often noticeably different from their high-latitude counterparts. Thus, while the underlying physiological regulation appears to be similar, low latitude species use torpor more flexibly, both in terms of seasonal timing and torpor bout length. Dausmann et al. explore aspects of the regulation of heterothermy in a tropical species, the lesser hedgehog tenrec (*Echinops telfairi*) that uses torpor throughout the year. Tenrecs use variation in environmental temperature to both passively rewarm from torpor (hence saving energy) and as a *zeitgeber* to synchronize active and resting periods during summer and to time arousals during hibernation. Flexible year-round use of heterothermy is also used by the edible dormouse (*Glis glis*). The seasonal adaptations of this species are reviewed by Ruf and Bieber. The edible dormouse not only adapt to predictable seasonal variation in climate, but also to semi-predictable year-to-year variation in food availability, and day-to-day variation in weather. Edible dormice can hibernate for almost a year and skip reproduction in resource-poor years but breed intensively and show hyperphagia in years when food is bountiful. The switch between these extreme strategies is modulated by future reproductive potential with increased risk-taking behavior as the animals age.

The use of heterothermy can also differ between sexes. Noiret et al. report that physiological, metabolic and behavioral responses to food shortage are more flexible in females compared to males of the gray mouse lemur (*Microcebus murinus*). This reiterates the notion that heterothermic responses are closely adjusted to both environmental and state-dependent variables (Boyles et al., 2007; Geiser, 2013). Apart from a direct role in starvation avoidance, heterothermy may also impact survival through a reduction in extrinsic (predation) and intrinsic (somatic wear) mortality risk (Nowack et al., 2017). Constant et al. explore some aspects of the evolution of hibernation behavior by investigating how longevity is affected by phylogenetic signals and activity-time budgets across a range of mammalian hibernators. The authors show that longevity increases with the duration of the hibernation season, particularly in small (<1.5 kg) hibernators. On the other hand, Landes et al. propose that a flexible use of heterothermy may carry ecological and physiological costs, such as changes to the neuroendocrine system and increased free-radical production. While these costs may be marginal at each transition event, they could accumulate over time and therefore emerge with age. Nevertheless, it is assumed that the flexible use of torpor will be beneficial when species need to adapt to climate change.

Global change and the associated increase in extreme weather events pose an increasing threat to living organisms. This is clearly shown by Weyer et al. in their study of aardvarks (*Orycteropus afer*). Aardvarks are nocturnal and tightly control their circadian body temperature rhythm under normal conditions. A summer drought was associated with reduced foraging activity, increased mortality and relaxed body temperature regulation of aardvarks. Individuals that died progressively switched to almost complete diurnal activity. This does not bode well for aardvarks facing climate change, nor for the many animals that depend on aardvark burrows for refuge. Roussel and Voituron express similar concerns for the common toad (*Bufo bufo*). Their study found that mitochondria in this species produce ATP less efficiently and at a higher oxidative cost during an acute heat challenge. This might require extra resources to maintain ATP production and oxidative balance, leaving less energy for other tasks during heat stress. In contrast, Cooper et al. show that free-ranging zebra finches (*Taeniopygia guttata*) can accommodate heatwaves without major impacts on energy or water turnover, provided that drinking water is available. The birds avoid or limit activity during the hottest part of the day and showed pre-emptive feeding and drinking in preparation for inactive periods. It seems that cold weather could be more challenging than heat waves for this desert bird. Stochastic weather events alter not only food and water access, but also availability of other components linked to energy metabolism, such as dissolved oxygen in aquatic systems. Marshall and McQuaid show that snails (*Turritella terebra*) balance restricted oxygen availability in hypoxic seawater by downregulating metabolism and energy allocation to various physiological functions. Yet, compensation was incomplete since the snails incurred an oxygen debt in severe hypoxia.

Climate change and extreme weather events affect not only mature life forms, but can have pronounced effects during development when embryonic/fetal endotherms are actually poikilothermic and ectotherms typically cannot thermoregulate behaviorally. This can shape energy-based trade-offs, such as between thermoregulation and growth and (later) between survival and reproduction (Nord and Nilsson, 2016; Andreasson et al., 2018). Hence, this Research Topic also explores effects of environmental fluctuations during development. Since genetic adaptation is often slow, there is growing interest in how behavioral and physiological flexibility helps mitigate the challenges of climate change since flexible phenotypes allow organisms to optimize energy allocation when conditions change. In line with this, Nord and Giroud have reviewed recent findings on the effects of developmental temperature in birds and mammals and discuss when the early thermal environment is predisposing or constraining for lifelong thermoregulatory performance in fluctuating environments, and if such effects are heritable. Stawski and Geiser manipulated developmental temperature in yellow-footed antechinus (*Antechinus flavipes*) and measured the effects on adult metabolic rates. The study suggests considerable phenotypic plasticity in both sexes, which is likely vital for a short-lived species that only breeds once in a lifetime. While phenotypic plasticity in response to fluctuating environmental conditions during development is often advantageous, so-called counter-gradient variation (Levins, 1968) can oppose environmental influences to reduce the extent of phenotypic adaptation. Pettersen reviews such effects on genetic and phenotypic levels in reptiles, concluding that counter-gradient variation in developmental time may be an important adaptive response to decrease developmental costs for animals in cool climates. These contributions indicate the advantages of phenotypic plasticity in response to environmental conditions and support the notion that the pressure of climate change will likely select for increased phenotypic plasticity rather than for directional adjustment of phenotypes (Canale and Henry, 2010).

Collectively, this Research Topic summarizes new and important insights into understanding how ecological and physiological adaptation allows animals to thrive when environments fluctuate seasonally or annually. It also reveals potential implications in terms of life histories of these efficient, sometimes limited, adaptive responses of animal species. We hope that this Research Topic will provide a solid platform from which to embark on multidisciplinary research endeavors that will be required to understand the challenges and capacities for adaptation to climate change in the twenty-first century and beyond.

## Author Contributions

SG drafted the editorial. JN, AN, and KS made critical comments and substantially edited the manuscript. All co-authors approved the final version of the editorial and agreed to be responsible for all contents.

## Conflict of Interest

The authors declare that the research was conducted in the absence of any commercial or financial relationships that could be construed as a potential conflict of interest.
